# Somatodendritic surface expression of epitope-tagged and KChIP binding-deficient Kv4.2 channels in hippocampal neurons

**DOI:** 10.1371/journal.pone.0191911

**Published:** 2018-01-31

**Authors:** Helena Prechtel, Sven Hartmann, Daniel Minge, Robert Bähring

**Affiliations:** Institut für Zelluläre und Integrative Physiologie, Zentrum für Experimentelle Medizin, Universitätsklinikum Hamburg-Eppendorf, Hamburg, Germany; University of South Alabama, UNITED STATES

## Abstract

Kv4.2 channels mediate a subthreshold-activating somatodendritic A-type current (I_SA_) in hippocampal neurons. We examined the role of accessory Kv channel interacting protein (KChIP) binding in somatodendritic surface expression and activity-dependent decrease in the availability of Kv4.2 channels. For this purpose we transfected cultured hippocampal neurons with cDNA coding for Kv4.2 wild-type (wt) or KChIP binding-deficient Kv4.2 mutants. All channels were equipped with an externally accessible hemagglutinin (HA)-tag and an EGFP-tag, which was attached to the C-terminal end. Combined analyses of EGFP self-fluorescence, surface HA immunostaining and patch-clamp recordings demonstrated similar dendritic trafficking and functional surface expression for Kv4.2[wt]^HA,EGFP^ and the KChIP binding-deficient Kv4.2[A14K]^HA,EGFP^. Coexpression of exogenous KChIP2 augmented the surface expression of Kv4.2[wt]^HA,EGFP^ but not Kv4.2[A14K]^HA,EGFP^. Notably, activity-dependent decrease in availability was more pronounced in Kv4.2[wt]^HA,EGFP^ + KChIP2 coexpressing than in Kv4.2[A14K]^HA,EGFP^ + KChIP2 coexpressing neurons. Our results do not support the notion that accessory KChIP binding is a prerequisite for dendritic trafficking and functional surface expression of Kv4.2 channels, however, accessory KChIP binding may play a potential role in Kv4.2 modulation during intrinsic plasticity processes.

## Introduction

Voltage-gated potassium (Kv) channels are critically involved in the control of excitability and in the shaping of action potentials in neurons and muscle cells. In many neurons members of the Kv4 subfamily, especially Kv4.2, mediate a subthreshold-activating somatodendritic A-type current (I_SA_; [1]). In hippocampal pyramidal neurons I_SA_ increases in amplitude with distance from the soma, counteracting dendritic excitation and suppressing back-propagating action potentials (b-APs; [2]). Moreover, I_SA_ attenuates dendritic excitatory postsynaptic potentials (EPSPs; [3]). On the other hand, Kv4.2 channel availability, and therefore I_SA_ and dendritic b-AP dynamics, can be modified in an activity-dependent manner, as observable in both epileptogenic disease models and models for long-term potentiation (LTP) induction [4–12]. Obviously, activity-dependent Kv4.2 modulation must be tightly controlled in order to significantly increase excitability locally, but without the loss of the protective "dendritic shock absorber" function [13] of Kv4.2-mediated I_SA_.

Kv4 channels may form complexes with two types of accessory β-subunits: membrane-spanning dipeptidyl aminopeptidase-like proteins (DPPs; [14]) and cytoplasmic Kv channel interacting proteins (KChIPs; [15]). There are four different KChIP subtypes (KChIP1, KChIP2, KChIP3 and KChIP4; [15, 16]) and two different DPP subtypes (DPP6 and DPP10, also referred to as DPPX and DPPY, respectively; [14, 17]), all capable of directly interacting with Kv4 channels. Native neuronal Kv4 channels may be formed by ternary complexes of Kv4 α-subunits, DPPs and KChIPs [18]. Coexpression studies in heterologous systems have shown that DPPs and most KChIPs, besides their specific effects on channel gating, enhance the functional surface expression of Kv4 channels, thereby dramatically increasing current amplitudes [14, 15, 19]. DPPs augment membrane trafficking of Kv4 channels and stabilize channel protein in the plasma membrane [17, 20, 21]. DPP6 seems to be critically involved in the generation of the somatodendritic I_SA_ gradient with enhanced Kv4.2 expression in distal dendrites of hippocampal neurons [2, 22]. Binding of KChIPs increases the number of Kv4 channels in the plasma membrane by various mechanisms, including promoted tetrameric assembly, a chaperone-like effect on membrane trafficking, increased protein stability and promoted surface retention [15, 19, 23–26]. Notably, KChIP2, KChIP3 and KChIP4 exhibit an interdependent synergy in stabilizing I_SA_ expression, and apparently compensate for one another, in cortical neurons [27], whereas in hippocampal neurons KChIP2 seems to be absolutely essential for the maintenance of normal excitability, with no functional redundancy [28]. Here, we asked whether the effects of KChIP binding on surface expression seen in heterologous expression systems may play a role in dendritic trafficking and functional surface expression of Kv4.2 channels in hippocampal neurons. Since KChIPs belong to the neuronal calcium sensor (NCS) family of Ca^2+^-binding proteins [29], potentially capable of linking neuronal Ca^2+^ influx to Kv4 channel function [30], we also asked whether KChIPs may play a role in the activity-dependent decrease in Kv4.2 channel availability. To answer these questions we expressed epitope-tagged and KChIP binding-deficient Kv4.2 channels in hippocampal neurons and studied their surface membrane expression with immunofluorescence and patch-clamp electrophysiology. Our results do not suggest that accessory KChIP binding is required for dendritic trafficking and functional surface expression of Kv4.2 channels, however, KChIPs may play a potential role in activity-dependent Kv4.2 channel modulation.

## Materials and methods

### Channel constructs

We used human Kv4.2 (AH009258) and human Kv channel interacting protein (KChIP) 2b (AF199598), referred to as KChIP2 below, in a pcDNA3 eukaryotic expression vector. Two epitopes were introduced in the Kv4.2 template: a hemagglutinin (HA)-tag (amino acid sequence in single letter code: YPYDVPDYA) was inserted in the extracellular loop between transmembrane segments S1 and S2, accessible for immunodetection from the external side (see below) and reporting surface membrane expression; and the complete enhanced green fluorescent protein (EGFP) sequence was fused to the C-terminal end (Kv4.2^HA,EGFP^; see S1A Fig). In addition, by PCR-based site-directed mutagenesis, the phenylalanine at position 11 or the alanine at position 14 (S1A Fig) were individually replaced by lysine to generate the constructs Kv4.2[F11K]^HA,EGFP^ and Kv4.2[A14K]^HA,EGFP^, respectively. These N-terminal point mutations have been previously shown to cause KChIP2 binding deficiency in Kv4.2 [31]. We also replaced the last 30 of the 630 amino acids of Kv4.2 (S1A Fig) by EGFP (Kv4.2[600Δ]^HA,EGFP^). For a similar Kv4.2 deletion mutant dendritic transport deficiency has been previously shown [32].

### Hippocampal primary culture and transfection

Kv4.2^HA,EGFP^ and KChIP2 cDNAs were expressed in hippocampal neurons. For this purpose hippocampal primary cultures were prepared from newborn Wistar rats, as described previously [33]. The preparation was carried out in strict accordance with institutional guidelines after approval from local authorities (Institutional Animal Care and Use Committee: Behörde für Gesundheit und Verbraucherschutz, Hamburg, Germany). The dissociated neurons were plated on poly-L-lysine-coated 12 mm glass coverslips and stored in 24-well plates (10^5^ cells per well) in Neurobasal-A medium with B27 supplement (Invitrogen) at 37°C and 5% CO_2_. After 7–9 days in culture the hippocampal neurons were transfected with the calcium phosphate technique [34], here described briefly with the amounts used per well: 2 μg total cDNA were dissolved in H_2_O (9 μl) and gently mixed with a 1M CaCl_2_ solutiuon (3 μl). After adding doubly concentrated HEPES-buffered saline (HBS, 12.5 μl) the transfection mix was incubated for 15 min. A large part (~80%) of the culture medium was removed from the well and stored, and the transfection mix (~25 μl) was given to the neurons. After 45 min at 37°C and 5% CO_2_ the transfection mix was removed, the cells were washed twice with HBS, and the original culture medium was returned to the well. Since the EGFP self-fluorescence (EGFP) of the Kv4.2^HA,EGFP^ constructs was too weak to be detected with the mercury lamp and filter system of the patch-clamp microscope, EGFP cDNA was cotransfected for all electrophysiology experiments to identify transfected neurons. All cotransfected cDNAs were used at equal amounts. No EGFP cDNA was cotransfected for confocal imaging experiments, and neurons transfected only with EGFP cDNA served as control.

### Immunocytochemistry and fluorescence measurements

The cultured hippocampal neurons were used for further experiments 1 or 2 days after transfection. All immunostainings, except the ones to detect the dendritic marker microtubule-associated protein (MAP) 2 (see below), were done with non-permeabilized cells. For this purpose the cultures were directly incubated with primary antibody (rat anti-HA, Roche; 1:500 in HBS, 20 min, 4°C), washed with phosphate-buffered saline (PBS) and fixed (HistoFix, Roth) for 12 min at room temperature (RT, 20–22°C). For MAP2 immunostaining the transfected neurons were fixed, washed with PBS and permeabilized with Triton X-100 (0.25% in PBS) for 3 min. After a 1 h blocking step with bovine serum albumine (BSA, 2% in PBS) the cells were incubated with primary antibody (rat anti-MAP2, Chemicon, 1:1000 in BSA solution; overnight, 4°C). Both non-permeabilized (anti-HA-stained) and permeabilized (anti-MAP2-stained) cultures were incubated in the dark with secondary antibody (anti rat Alexa-546 from goat, Invitrogen, 1:2500 in BSA solution; 1–2 h, RT), washed with PBS and mounted onto microscope slides. MAP2-related immunofluorescence (α-MAP2), HA-related surface immunofluorescence (α-HA surface), and EGFP were detected with a TCS SP2 confocal laser scanning microscope (Leica) in sequential line scan mode using LCS lite software (Leica). Identical laser and gain settings were used throughout the study. For α-MAP2, α-HA surface and EGFP, intensity profiles were obtained along defined portions of dendritic branches (one per cell). Background noise was subtracted, and HA signals without a corresponding EGFP signal were excluded from the analysis.

### Electrophysiology

Recordings from hippocampal neurons were performed at RT with the somatic whole-cell patch-clamp technique, as described previously [35]. The neurons were bathed in a solution containing (in mM) 160 NaCl, 2.5 KCl, 2 CaCl_2_, 1 MgCl_2_, 10 HEPES, 10 glucose (pH 7.3, NaOH). The pipette solution contained (in mM) 140 K-MeSO_3_, 5 KCl, 0.5 CaCl_2_, 0.5 MgCl_2_, 5 EGTA, 10 HEPES, 2 Mg-ATP (pH 7.2, KOH). For the recordings pyramidally shaped neurons [35, 36] with EGFP fluorescence were chosen. Recordings were done with an EPC9 patch-clamp amplifier controlled by Pulse software (HEKA). The liquid junction potential error (-7 mV in the pipette) was corrected online, and series resistance (6–15 MΩ) compensation was usually between 50 and 75%. The holding voltage was -70 mV, and outward currents were evoked by voltage pulses to +40 mV from a prepulse voltage of either -110 mV (activation of compound outward current) or -30 mV (I_SA_ component inactivated). The -30 mV prepulse protocol was followed by a P/3 protocol for offline leak subtraction (see below).

### Stimulation of synaptic activity

For some experiments neurons coexpressing Kv4.2[wt]^HA,EGFP^ + KChIP2 or Kv4.2[A14K]^HA,EGFP^ + KChIP2 were treated with α-amino-3-hydroxy-5-methyl-4-isoxazolepropionic acid (AMPA, 100 μM) to stimulate synaptic activity, as described previously [6]. AMPA treatment took place one day after transfection. The neurons were incubated (37°C and 5% CO_2_) with the AMPA-containing culture medium for 12 min, and electrophysiological recordings were performed in a time window of 5–60 min after the treatment.

### Data analysis

Fluorescence intensity profiles were taken as a measure of surface expression (α-HA surface) and total expression (EGFP) of Kv4.2^HA,EGFP^ for a defined portion of dendritic branch. Integrals under the α-HA surface and the EGFP intensity profiles were calculated for each analysed portion of dendritic branch (i.e., for each cell), and the integral ratio α-HA surface / EGFP (%) was taken as a measure of the fractional Kv4.2^HA,EGFP^ surface expression within that portion of dendritic branch. Current traces were analysed with PulseFit (HEKA). From the -110 mV prepulse current trace the corresponding -30 mV prepulse current trace was subtracted to obtain the I_SA_ component. The non- or slowly inactivating delayed rectifier current component (I_D_; [37]) was obtained by leak-subtracting the -30 mV prepulse trace. For both I_SA_ and I_D_ the peak amplitude was measured, and the ratio I_SA_ / I_D_ was determined (to adapt I_SA_ magnitude for neurons with different compound current expression levels). Graphing and statistical analyses of the data were done with Kaleidagraph (Synergy Software) and Prism (GraphPad), and pooled data are presented as mean ± SEM. Statistical significance of the differences between two groups was examined with Student’s t-test (see S2 Table). For multiple groups one-way analysis of variance (ANOVA) with Bonferroni post-hoc testing was used (see S1 Table).

## Results

### Dendritic trafficking and surface expression of epitope-tagged Kv4.2 protein

**Fig 1:**
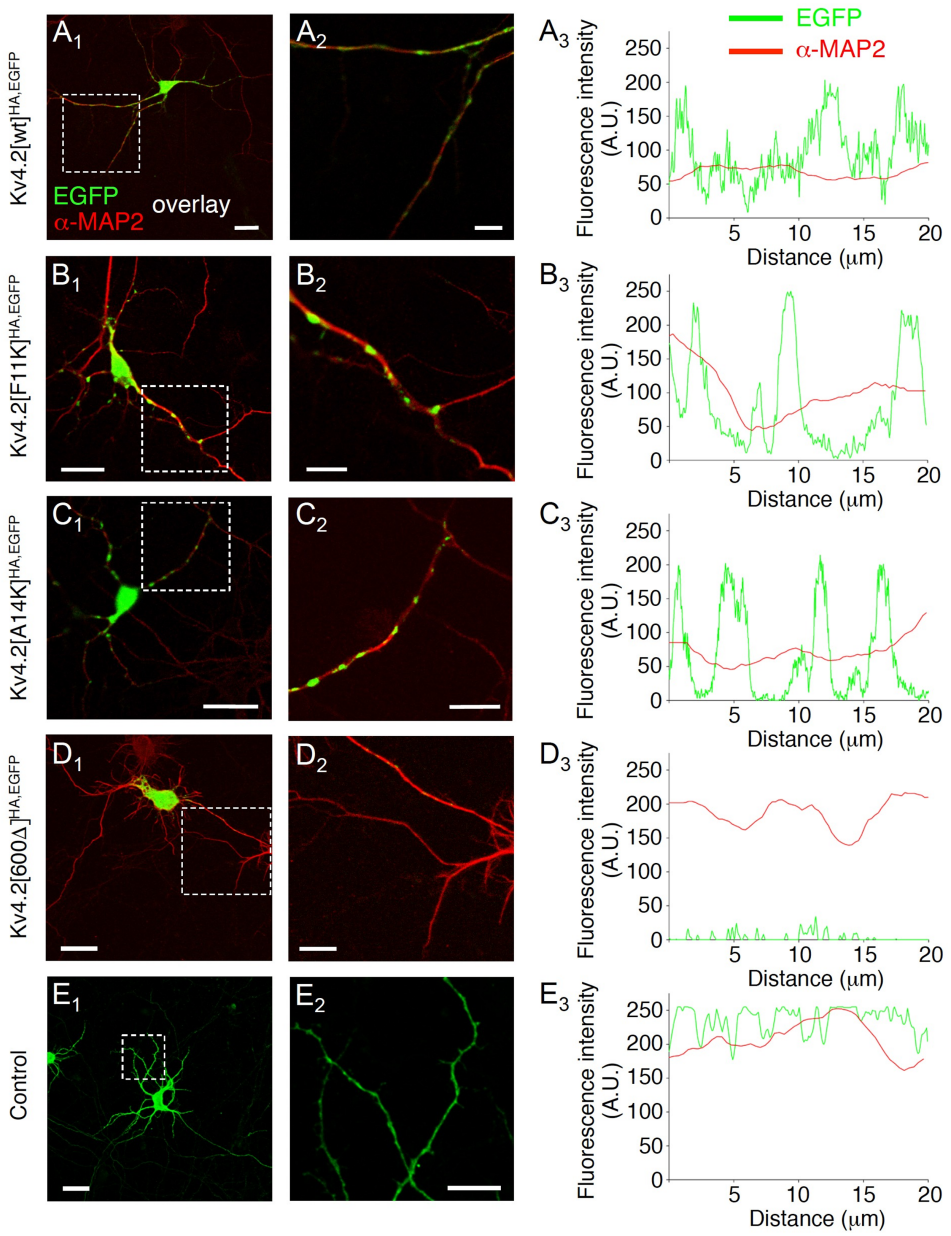
KChIP-independent trafficking of Kv4.2^HA,EGFP^ into dendrites. Cultured hippocampal neurons were transfected with cDNAs coding for different Kv4.2^HA,EGFP^ constructs, and their EGFP self-fluorescence (EGFP) was compared to MAP2 immunostaining (α-MAP2). **A**_**1**_**.** Overlay fluorescence image of a Kv4.2[wt]^HA,EGFP^ expressing neuron; **A**_**2**_**.** Enlarged view of the boxed area in A_1_; **A**_**3**_**.** Fluorescence intensity profile for EGFP (green) and α-MAP2 (red) for a portion of dendritic branch of the Kv4.2[wt]^HA,EGFP^ expressing neuron. **B**_**1**_**—B**_**3**_**.** Overlay fluorescence images and fluorescence intensity profile along a portion of dendritic branch of a Kv4.2[F11K]^HA,EGFP^ expressing neuron. **C**_**1**_**—C**_**3**_**.** Overlay fluorescence images and fluorescence intensity profile along a portion of dendritic branch of a Kv4.2[A14K]^HA,EGFP^ expressing neuron. **D**_**1**_**—D**_**3**_**.** Overlay fluorescence images and fluorescence intensity profile along a portion of dendritic branch of a Kv4.2[600Δ]^HA,EGFP^ expressing neuron. **E**_**1**_**—E**_**3**_**.** EGFP fluorescence images and fluorescence intensity profile along a portion of dendritic branch of a control neuron (transfected only with EGFP cDNA). Scale bars in vertical column A_1_—E_1_: 25 μm; scale bars in vertical column A_2_—E_2_: 10 μm.

In order to define the role of KChIP binding in dendritic trafficking of Kv4.2 protein in a native environment we decided to express epitope-tagged and KChIP binding-deficient Kv4.2 constructs in cultured hippocampal neurons. Our Kv4.2^HA,EGFP^ constructs (S1A Fig) are well suited for this application: As Western and coimmunoprecipitation analyses showed beforehand, the HA epitope is reliably detected by the respective antibody, and the EGFP epitope neither compromizes protein expression (S1B Fig), nor changes the known KChIP2 binding properties of Kv4.2[wt]^HA^ and Kv4.2[A14K]^HA^ (S1C Fig; [31]). Moreover, α-HA surface and EGFP intensity profiles nicely reflect surface and total protein, respectively, in a cellular environment (S2 Fig). The experiments done in hippocampal primary cultures showed that Kv4.2[wt]^HA,EGFP^ formed clusters in the dendritic branches defined by the α-MAP2 signal (Fig 1A). Notably, the KChIP binding deficiency mutants Kv4.2[F11K]^HA,EGFP^ (Fig 1B) and Kv4.2[A14K]^HA,EGFP^ (Fig 1C) showed a similar subcellular distribution as Kv4.2[wt]^HA,EGFP^. By contrast, the dendritic transport deficiency mutant Kv4.2[600Δ]^HA,EGFP^ [32] showed no α-MAP2 colocalization, but was retained in the soma (Fig 1D), and EGFP expressed alone was evenly distributed in both soma and dendrites (Fig 1E; see Discussion). Our data suggest that accessory KChIP binding is not a prerequisite for the trafficking of Kv4.2 protein into dendrites.

**Fig 2:**
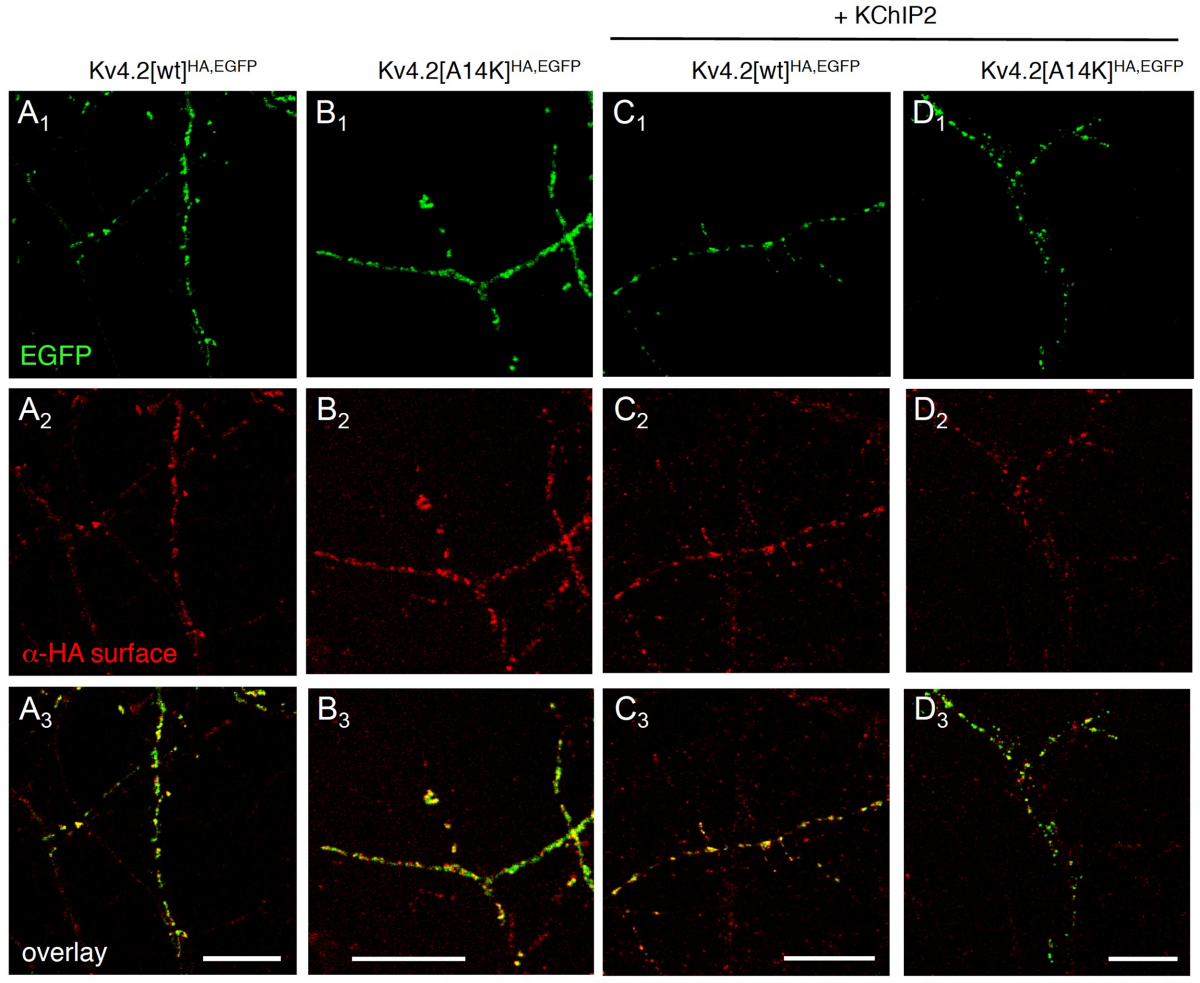
KChIP-independent surface expression of Kv4.2^HA,EGFP^ in dendrites. Neurons were transfected with cDNAs coding for Kv4.2[wt]^HA,EGFP^ or the KChIP binding-deficient mutant Kv4.2[A14K]^HA,EGFP^, in the absence or presence of KChIP2 cDNA. EGFP self-fluorescence (EGFP) and HA surface immunostaining (α-HA surface) reflect total and surface expression of Kv4.2^HA,EGFP^, respectively. Colocalization of EGFP and α-HA surface was assessed from the merged pictures (overlay). **A**_**1**_**—A**_**3**_**.** Dendritic branches of a Kv4.2[wt]^HA,EGFP^ expressing neuron. **B**_**1**_**—B**_**3**_**.** Dendritic branches of a Kv4.2[A14K]^HA,EGFP^ expressing neuron. **C**_**1**_**—C**_**3**_**.** Dendritic branches of a neuron coexpressing Kv4.2[wt]^HA,EGFP^ + KChIP2. **D**_**1**_**—D**_**3**_**.** Dendritic branches of a neuron coexpressing Kv4.2[A14K]^HA,EGFP^ + KChIP2. Horizontal row A_1_—D_1_: EGFP; horizontal row A_2_—D_2_: α-HA surface; horizontal row A_3_—D_3_: overlay (merged pictures); scale bars: 15 μm.

**Fig 3:**
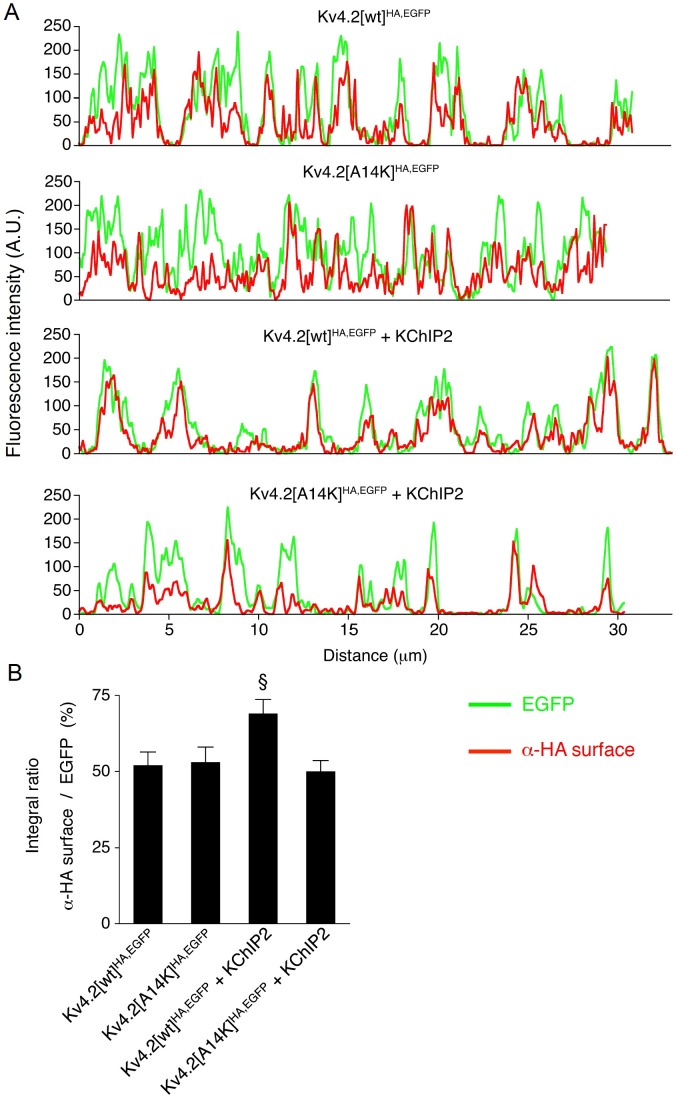
Relative surface expression of Kv4.2^HA,EGFP^. Quantification of relative surface expression based on fluorescence intensity profiles for EGFP self-fluorescence (EGFP) and surface HA immunostaining (α-HA surface). **A.** Expression profiles along a portion of dendritic branch of roughly 30 μm from 4 different neurons expressing Kv4.2[wt]^HA,EGFP^, Kv4.2[A14K]^HA,EGFP^, Kv4.2[wt]^HA,EGFP^ + KChIP2 or Kv4.2[A14K]^HA,EGFP^ + KChIP2, respectively. **B.** Integral ratios (see Materials and methods). Note that relative surface expression was significantly increased for Kv4.2[wt]^HA,EGFP^ (§, one-way ANOVA), but not for Kv4.2[A14K]^HA,EGFP^, by the coexpression of exogenous KChIP2.

We next asked whether KChIP binding is necessary for surface expression of Kv4.2 and examined α-HA surface signals (Fig 2). Both Kv4.2[wt]^HA,EGFP^ (Fig 2A) and Kv4.2[A14K]^HA,EGFP^ expressing neurons (Fig 2B) showed not only their inherent EGFP signal but also a clear α-HA surface signal, suggesting that either construct is inserted into the surface membrane. Visual inspection of the merged pictures (overlay of EGFP and α-HA surface) allowed already an estimate of the fractional surface expression. It was obvious that not all of the heterologously expressed channel protein present in dendrites was inserted in the surface membrane (Fig 2A_3_ and 2B_3_). Due to the overexpression of Kv4.2^HA,EGFP^ the transport machinery of the cells may have been saturated, which would be critical for the interpretation of our experimental results. Therefore we checked whether the fractional surface expression of Kv4.2^HA,EGFP^ could be still increased by coexpressing exogenous KChIP2 (Fig 2C and 2D). In these coexpression experiments the merged pictures suggested, indeed, that Kv4.2[wt]^HA,EGFP^ surface expression was more efficient in the presence of exogenous KChIP2 (Fig 2C_3_), whereas this was not obvious in neurons coexpressing Kv4.2[A14K]^HA,EGFP^ + KChIP2 (Fig 2D_3_). We analysed our fluorescence images based on intensity profiles along portions of dendritic branches (Fig 3). From the examples shown in Fig 3A it becomes obvious that the one-to-one correspondence between EGFP and α-HA surface intensity peaks, which was moderate for the Kv4.2[wt]^HA,EGFP^ expressing and the Kv4.2[A14K]^HA,EGFP^ expressing neuron, was strongly increased in the Kv4.2[wt]^HA,EGFP^ + KChIP2 coexpressing, but not in the Kv4.2[A14K]^HA,EGFP^ + KChIP2 coexpressing neuron. We quantified these observations for a large number of neurons by generating integral ratios for the dendritic branches analysed (see Materials and methods; Fig 3B). The data are summarized in S1 Table. Although aware that our fluorescence signals cannot be directly translated into amount of protein, the results suggested that within the analysed portions of dendritic branches the fractional surface expression of Kv4.2[wt]^HA,EGFP^, but not of Kv4.2[A14K]^HA,EGFP^, was significantly inceased from ~50% to ~70% (1.33-fold) by excess supply of exogenous KChIP2 (see Discussion).

### Dynamic I_SA_ expression in transfected hippocampal neurons

**Fig 4:**
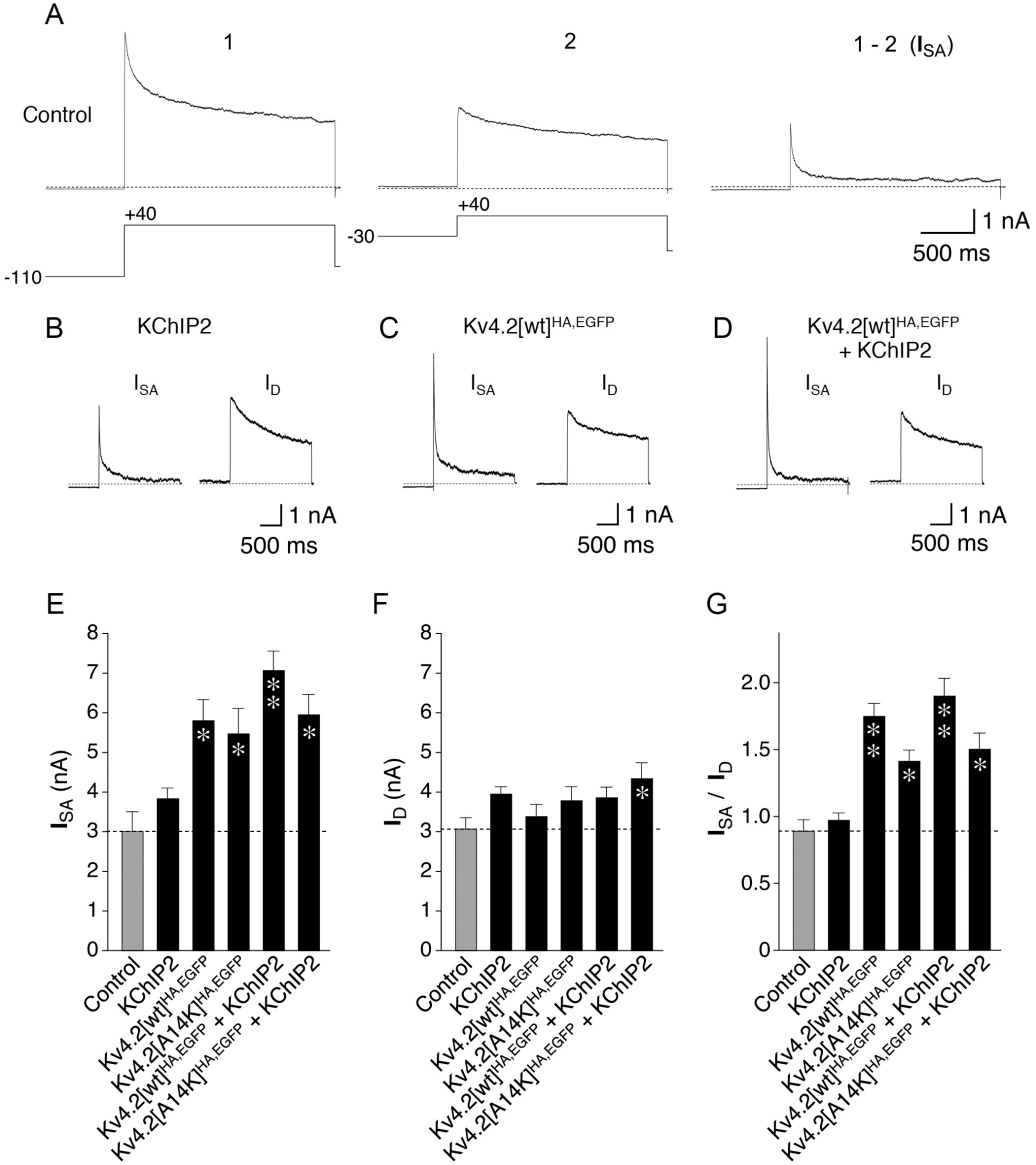
I_SA_ and I_D_ amplitudes in transfected hippocampal neurons. Currents were recorded with the patch-clamp technique in the somatic whole-cell configuration. **A.** The prepulse-inactivation-subtraction method was used to isolate I_SA_ (see Materials and methods). Voltage protocols shown below traces **B.** I_SA_ and I_D_ components of a hippocampal neuron transfected (in addition to EGFP) only with KChIP2 cDNA; **C.** I_SA_ and I_D_ components of a hippocampal neuron transfected with Kv4.2[wt]^HA,EGFP^ cDNA; **D.** I_SA_ and I_D_ components of a hippocampal neuron cotransfected with Kv4.2[wt]^HA,EGFP^ and KChIP2 cDNA; **E.** I_SA_ amplitudes; **F.** I_D_ amplitudes; **G.** I_SA_ / I_D_ ratios (to adapt I_SA_ magnitude for neurons with different compound current expression levels); * significantly different from control with 0.0001 ≤ p < 0.05; ** significantly different from control with p < 0.0001 (one-way ANOVA).

To test whether our α-HA surface signal reflected the membrane insertion of functional channels we performed whole-cell patch-clamp recordings on the transfected neurons (Fig 4). The I_SA_ component was isolated by the prepulse-inactivation-subtraction method (Fig 4A, see Materials and methods) and compared to the non- or slowly inactivating I_D_ component (examples shown in Fig 4B, 4C and 4D). In control neurons (expressing only EGFP) the mean I_SA_ amplitude was 3.01 nA (Fig 4E), the mean I_D_ amplitude 3.08 nA (Fig 4F), and pairwise calculations for each individual neuron yielded a mean I_SA_ / I_D_ ratio of 0.891 (Fig 4G). The results of our current measurements are summarized in S1 Table. Notably, if neurons were transfected (in addition to EGFP) only with KChIP2 cDNA, there was a trend towards larger amplitudes, but no significant increase in either current component (Fig 4B, 4E and 4F), and the mean I_SA_ / I_D_ ratio was 0.974 (Fig 4G). With all other transfections the I_SA_ amplitude was significantly larger than control (Fig 4E), which did not apply to the I_D_ amplitude (except for Kv4.2[A14K]^HA,EGFP^ + KChIP2, Fig 4F). Accordingly, the I_SA_ / I_D_ ratios obtained with these transfections were all significantly larger than control (between 1.416 and 1.902; Fig 4G, S1 Table). Notably also, neither for Kv4.2[A14K]^HA,EGFP^ (I_SA_ 1.09-fold; currents not shown) nor for Kv4.2[wt]^HA,EGFP^ (I_SA_ 1.22-fold; Fig 4C and 4D) the coexpression of exogenous KChIP2 led to a significant increase in I_SA_ or the I_SA_ / I_D_ ratio (Fig 4E and 4G). The absence of a significant I_SA_ increase in Kv4.2[wt]^HA,EGFP^ + KChIP2 coexpressing neurons, which is not in accordance with a clear increase in the corresponding α-HA surface / EGFP integral ratio (Fig 3B), may be due to the fact that somatic rather than dendritic I_SA_ dominated our patch-clamp recordings. We performed nucleated-patch recordings to get an impression of the relative contribution of somatic I_SA_. The results suggested that our whole-cell recordings comprise a significant fraction of dendritic I_SA_ (S4 Fig), but probably not enough to reflect I_SA_ expression in all distal dendrites. Despite this shortcoming, our electrophysiology results support the notion that KChIP binding is not a prerequisite for the insertion of functional Kv4.2^HA,EGFP^ channels in the surface membrane, albeit there was a trend towards larger amplitudes for Kv4.2[wt]^HA,EGFP^ compared to Kv4.2[A14K]^HA,EGFP^ both in the absence and presence of exogenous KChIP2 (Fig 4G, S1 Table; see Discussion).

**Fig 5:**
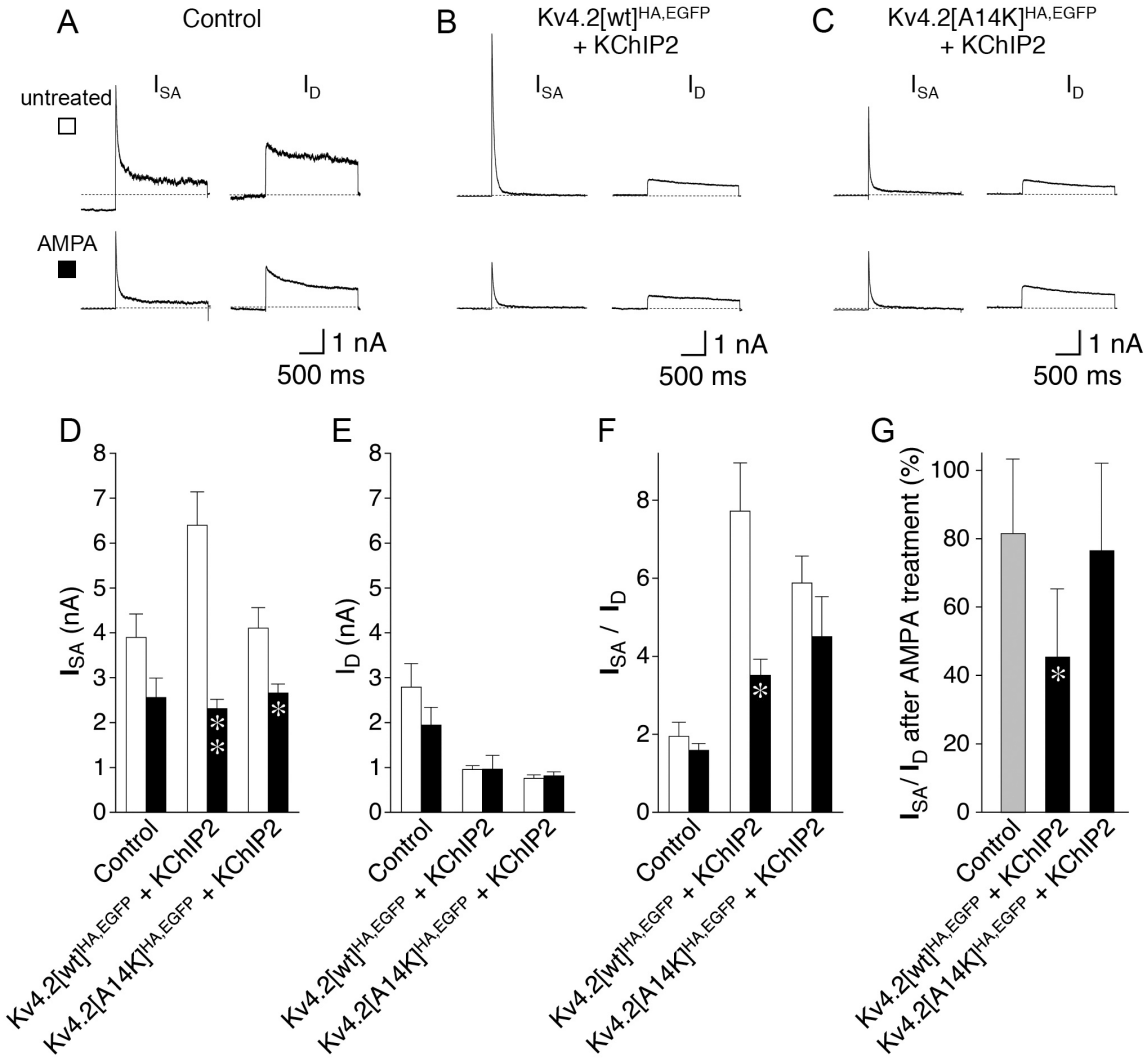
Activity-dependent modulation of I_SA_. Transfected hippocampal primary cultures were treated with AMPA (see Materials and methods) prior to recording. Whole-cell patch-clamp experiments were performed, and I_SA_ and I_D_ components were isolated. Representative current traces for untreated (empty symbol) and AMPA-treated neurons (black symbol) are shown for control (expressing only EGFP, **A**), Kv4.2[wt]^HA,EGFP^ + KChIP2 (**B**) and Kv4.2[A14K]^HA,EGFP^ + KChIP2 (**C**). **D.** I_SA_ amplitudes; **E.** I_D_ amplitudes; **F.** I_SA_ / I_D_ ratios (to adapt I_SA_ magnitude for neurons with different compound current expression levels); empty bars: untreated neurons; black bars AMPA-treated neurons. **G.** The remaining I_SA_ / I_D_ ratio following AMPA treatment (unrelated groups) is shown in %; * significantly different from untreated neurons (Student’s t-test).

It has been shown previously, that Kv4.2 channels are internalized in a Ca^2+^-dependent manner when synaptic activity is strongly stimulated by different methods [6]. Here, we chose AMPA treatment ([6]; see Materials and methods) to induce such modulation in our transfected hippocampal neurons. In particular, we asked whether KChIP-bound Kv4.2^HA,EGFP^ channels may react differently to the treatment with AMPA than KChIP-unbound ones. To this end, we performed the AMPA treatment with control, Kv4.2[wt]^HA,EGFP^ + KChIP2 coexpressing and Kv4.2[A14K]^HA,EGFP^ + KChIP2 coexpressing neurons, and analysed I_SA_ and I_D_ amplitudes as well as I_SA_ / I_D_ ratios (Fig 5 and S2 Table). Different from control (Fig 5A and 5D, see Discussion), we found significantly smaller I_SA_ amplitudes for both Kv4.2[wt]^HA,EGFP^ + KChIP2 (Fig 5B and 5D) and Kv4.2[A14K]^HA,EGFP^ + KChIP2 (Fig 5C and 5D) if the neurons had been treated with AMPA, but no changes in I_D_ amplitudes (Fig 5B, 5C and 5E). Notably, analysis of I_SA_ / I_D_ ratios revealed a stronger AMPA-induced decrease (fraction remaining ~40% instead of ~80%) exclusively for the Kv4.2[wt]^HA,EGFP^ + KChIP2 coexpressing neurons (Fig 5F and 5G). Taken together, our results support the notion that KChIP binding, although not a prerequisite for functional somatodendritic surface expression, may play a potential role in the activity-dependent modulation of Kv4.2 channels.

## Discussion

We have studied the somatodendritic surface expression of epitope-tagged and KChIP binding-deficient Kv4.2 channels exogenously expressed in cultured hippocampal neurons. Our results do not suggest that KChIP binding to Kv4.2 is a prerequisite for trafficking into dendrites and functional surface expression of the channels. The results further suggest that KChIP2-bound Kv4.2 channels may be more prone to an activity-dependent form of modulation, that leads to decreased channel availability.

### Exogenous expression of tagged Kv4.2 mutant constructs

Tagging ion channel proteins with foreign epitopes allows their easy and/or reliable detection, especially in cellular environments where endogenous forms of the respective protein are expressed. Detection is either based on fluorescent epitopes, or on highly epitope-specific binding partners such as antibodies or toxins [38]. Epitope-tagging has also been exploited to study the subcellular targeting and trafficking of Kv4.2 channels in neurons. Kv4.2 constructs with green or cyan fluorescent protein (GFP or CFP, respectively), or the mCherry amino acid sequence attached to the N- or C-terminal end, have allowed live cell imaging to study Kv4.2 dendritic trafficking and activity-dependent redistribution ([6, 39–41]; see below). Alternatively, the myc antigen [42] or the α-bungarotoxin binding domain [43] have been introduced between S1 and S2 of Kv4.2 in order to study subcellular targeting and to measure surface expression. The results obtained with epitope-tagged ion channel constructs must be interpreted with caution, since the epitope may interfere not only with channel gating, but also with targeting and trafficking [38]. Here we used a combination of C-terminal EGFP-tagging and S1-S2 HA-tagging in order to simultaneously assess total and surface expression, respectively, of Kv4.2. Biochemical and electrophysiological experiments in CHO cells validated that our Kv4.2^HA,EGFP^ constructs, like the previously studied Kv4.2^HA^ constructs [31] and untagged Kv4.2 channels [19], expressed normally and mediated typical A-type currents subject to modulation by KChIP2 binding, unless they carried the A14K mutation. Our data are in accordance with previous results demonstrating that neither N-terminal nor C-terminal attachment of GFP in Kv4.3 interferes with KChIP1 interaction [44, 45]. Most importantly, our Kv4.2^HA,EGFP^ constructs formed dendritic clusters, yielding pictures similar to the immunostaining of endogenous Kv4.2 [26, 46, 47], and it has been previously shown by others that exogenous EGFP-tagged Kv4.2 constructs, like their native counterparts [48], are enriched at points of synaptic contact (i.e., dendritic spines; [6, 40]). However, in the absence of data based on a suitable marker we cannot judge whether our Kv4.2^HA,EGFP^ constructs are enriched in dendritic spines.

To study the roles of accessory DPPs and KChIPs in neuronal Kv4 channel function, corresponding knockout models [22, 27, 28] have been used. Here we pursued a different strategy, by expressing accessory subunit binding-deficient Kv4.2 mutants in neurons. Under the reasonable assumption that the Kv4.2 structural determinants identified for KChIP2 binding [31] also apply to other KChIPs, the Kv4.2[A14K]^HA,EGFP^ and Kv4.2[F11K]^HA,EGFP^ mutants used in the present study allow the assessment of the role played by KChIP binding in the fate and function of Kv4.2 channels. The approach does not interfere with other KChIP functions unrelated to Kv4.2, however, one should keep in mind that transient cDNA plasmid-based overexpression of ion channels certainly differs from native gene expression, and therefore, the results must be interpreted with great care. In particular, due to the overexpression of channel protein the transport machinery of the cell may be overloaded, which harbors the risk of a ceiling effect (see below).

### Control of Kv4.2 channel targeting, trafficking and surface abundance

Many neuronal ion channel proteins exhibit polarized subcellular sorting; i.e., they are predominantly found either in axonal or in dendritic compartments. In search of a dendritic localization signal in the Kv4.2 primary structure Rivera and coworkers identified a stretch of amino acids in the C-terminus (amino acids 474–489) conserved in all Shal-related channels and containing a dileucine motif (LL), which conforms to the canonical EXXXXXLL sequence [42, 49]. However, it turned out that the actual dendritic transport is controlled by a stretch of 30 amino acids at the C-terminal end of Kv4.2 (see S1A Fig), which mediates an interaction with the kinesin transport protein Kif17 [32]. We have previously identified not only N-terminal but also C-terminal Kv4.2 domains involved in KChIP interaction, including a stretch between amino acids 478 and 490 [31], which covers the dileucine motif, but not the distal trafficking domain. Since the dileucine motif has been shown to be both necessary (in Kv4.2) and sufficient (in Kv1-LL and CD8-LL mutant constructs) for dendritic targeting [42], we considered it unlikely that KChIP binding plays a role in polarized subcellular sorting, comparable to the reported necessity of Kvβ-subunit binding for axonal Kv1 channel targeting [50]. However, since the functional interdependence of the dileucine targeting motif and the distal transport domain of Kv4.2 has not been resolved so far [32, 42], we initially considered the possibility that KChIP binding may somehow be involved in dendritic trafficking. Although intriguing, this hypothesis was not supported by our data, which showed a similar dendritic clustering for wild-type and the KChIP binding-deficient Kv4.2 mutants. Artificial overexpression and saturation of the transport machinery may have caused an unspecific distribution, allowing the KChIP binding-deficient Kv4.2 mutants to diffuse into dendrites. However, as shown by our experiments, free diffusion of protein, although possible (in control neurons expressing only EGFP), is not seen for Kv4.2 channel constructs, which, even if overexpressed, are reliant on trafficking signals (as shown by the somatic retention of Kv4.2[600Δ]^HA,EGFP^; see Fig 1 and [32]). Moreover, the finding that wild-type surface expression could be increased by adding exogenous KChIP2 suggests that Kv4.2^HA,EGFP^ surface expression had not been saturated due to artificial overexpression in the first place. It should be noted that our N-terminal point mutations (F11K and A14K), although preventing stable Kv4.2/KChIP complex formation, left the C-terminal interaction sites [31] intact, maybe allowing for transient or loose KChIP association, which may be important for Kif17 action [32]. However, in the absence of appropriate immunocytochemical data to answer these questions, there is no evidence to support the notion that KChIP binding plays a significant role in Kv4.2 channel targeting and trafficking.

Kv4/KChIP channels also mediate a transient outward current (I_to_) in cardiomyocytes [51]. From its known role in defining a transmural I_to_ gradient in cardiac tissues [52], one may, on a cellular level, ascribe an involvement in the generation of the somatodendritic I_SA_ gradient to KChIPs, but we have no data to support or exclude this hypothesis. From their finding that the somatodendritic I_SA_ garadient is lost in DPP6 knockout mice, Sun and coworkers concluded that Kv4.2/DPP6 association must be critically involved [22]. Notably, these authors not only observed reduced Kv4.2 expression but also reduced KChIP expression in distal dendrites in the DPP6 knockout mice [22], reminiscent of the reduced KChIP expression found in Kv4.2 knockout mice [53, 54] and corroborating the notion that the expression levels of Kv4.2 and KChIP are tightly coupled. KChIP knockout models have shown that KChIP2 plays a critical role in controlling I_SA_, with a lack of redundancy among different KChIPs, in hippocampal neurons [28], and that in cortical neurons the remaining KChIPs are upregulated if one KChIP gene is specifically deleted [55]. The apparent necessity of KChIPs for normal I_SA_ expression, suggested by these results, contradicts our experimental findings, which suggest that KChIP binding is not a prerequisite for Kv4.2 surface expression. Of note, in our experiments none of the native genes (Kv4.2 or KChIP) was deleted and additional exogenous Kv4.2 cDNA was provided with the transfection. Our results demonstrate that the use of binding-deficient channel mutants yields valuable information in addition to the data obtained with accessory subunit knockout, by circumventing possible indirect side effects caused by gene deletion and/or by the complete absence of a protein. We also introduced mutations at sites homologous to the ones previously reported for Kv4.3 and DPP10 interaction [56] but failed to induce DPP interaction deficiency in Kv4.2 (not shown).

### Activity-dependent modulation of Kv4.2 channels

Activity-dependent intrinsic plasticity in neurons, be it physiological (e.g., during LTP induction) or pathophysiological (e.g., during status epilepticus), is associated with ion channel modulation, usually as a consequence of massive Ca^2+^ influx and kinase activation. Ion channel modulation may consist in changes in biophysical properties, like the voltage dependencies of activation or inactivation, changes in the number of channels, or both. Such an activity-dependent modulation of Kv4.2 channels is reflected for instance by the finding that the voltage dependence of I_SA_ inactivation is negatively shifted, in an NMDA receptor-dependent manner, after LTP induction, leading to a decrease in I_SA_ availability and enhanced local excitability in CA1 pyramidal dendrites [5]. I_SA_ has also been shown to be downregulated (and dendritic excitability increased) by pharmacological activation of protein kinase A (PKA) or protein kinase C (PKC), which led to a positive shift in the voltage dependence of I_SA_ activation [57, 58]. In vitro stimulation of synaptic activity by different methods (including AMPA treatment, as in the present study) has been shown to cause clathrin-mediated endocytosis of Kv4.2 channels and their trafficking out of spines, as well as a reduction in total Kv4.2 protein, in an NMDA receptor and Ca^2+^-dependent manner [6, 7]. Closer inspection revealed that these processes depend on PKA-mediated phosphorylation of Kv4.2 at Ser 522 [59], with an involvement of A kinase anchoring protein (AKAP) 79/150, and that the phosphatase calcineurin counteracts Kv4.2 internalization [60]. Very similar to the processes described above, status epilepticus (SE) induction leads to reduced b-AP attenuation in CA1 pyramidal neurons due to a reduction in total Kv4.2 protein and Kv4.2 mRNA, and the remaining Kv4.2 channels were found to be strongly phosphorylated by extracellular signal-regulated kinase (ERK), presumably downstream of PKA and/or PKC activation [4, 8, 61]. In the present study we adopted the method of AMPA treatment from Kim and coworkers [6] to stimulate synaptic activity, and it is likely that the I_SA_ amplitude differences observed in our experiments are, at least in part, due to Kv4.2 channel internalization. Notably, the remaining I_SA_ amplitudes after AMPA treatment were more or less identical for all of our experimental settings (control, Kv4.2[wt]^HA,EGFP^ + KChIP2 and Kv4.2[A14K]^HA,EGFP^ + KChIP2), suggesting that there may be a pool of Kv4.2 channels resistant to internalization under our experimental conditions. While Kim and coworkers observed a ~ 24% reduction [6], we observed a ~ 34% reduction in the native I_SA_ amplitude (control), which, however, failed to reach significance (see Fig 5D). The absence of a significant effect on total Kv4.2 protein reported by Lei and coworkers [7], when using the same method, may have been due to the relatively low AMPA concentration used by these authors. Despite these experimental complications, our data suggest that KChIP2 bound to Kv4.2 makes the channels more prone to activity-dependent modulation. We found that the difference between the I_SA_ amplitudes measured in untreated and AMPA-treated Kv4.2[wt]^HA,EGFP^ + KChIP2 expressing cells was more pronounced than in cells expressing the KChIP binding-deficient mutant Kv4.2[A14K]^HA,EGFP^ + KChIP2 (see Fig 5D), and normalized to I_D_ the difference caused by AMPA treatment was significant only in the Kv4.2[wt]^HA,EGFP^ + KChIP2 expressing cells (see Fig 5F and 5G). It is intriguing that both DPP6 and KChIPs have been shown to make Kv4.2 channels sensitive to PKA-mediated phosphorylation [21, 62]. If activity-dependent modulation, including internalization, is dependent on PKA-mediated phosphorylation, then KChIP-bound Kv4.2 channels (i.e., wild-type but not our A14K mutant) are expected to be more prone to this form of modulation, a notion which is supported by our data. Also, Kim and coworkers reported faster I_SA_ decay following AMPA treatment and suspected a subset of Kv4.2 channels being internalized [6]. These internalized Kv4.2 channels may have been the ones with slower inactivation kinetics due to KChIPs bound to their N-termini [19]. However, in the present study we did not observe a significant difference between the I_SA_ decay kinetics in untreated and AMPA-treated control neurons (not shown).

In summary, the combined findings suggest that accessory KChIPs are not a prerequisite for dendritic Kv4.2 channel targeting and trafficking. An involvement of KChIPs, together with DPP6, in the generation of the somatodendritic I_SA_ gradient has not been conclusively clarified. In addition to acting as a Ca^2+^ sensor for acute Kv4 channel gating modulation [30, 63], KChIPs may control activity- and kinase-dependent intrinsic neuronal remodeling processes involving Kv4.2 channel modulation and/or internalization. Whether the Ca^2+^ binding properties of KChIPs [29] play a role in the latter processes, remains to be determined.

## Supporting information

S1 Fig Immunochemical detection and KChIP2 binding properties of epitope-tagged Kv4.2 protein. Kv4.2^HA,EGFP^, Kv4.2^HA^ and Kv4.3^HA^ constructs were coexpressed with KChIP2 in Chinese hamster ovary (CHO) cells in different combinations and detected with HA- and panKChIP-antibodies (see S1 Methods). A. Membrane topology model of the Kv4.2^HA,EGFP^ α-subunit. External HA-tag inserted between transmembrane segments S1 and S2 and EGFP attached to the C-terminal end. Phenylalanine (Phe) 11 and alanine (Ala) 14 near the cytoplasmic N-terminus, and the C-terminal region between amino acid residue 600 and 630, relevant for dendritic transport [32], are indicated. B. Transfected cDNAs (lanes 1–9) and corresponding Western blot analysis (WB) with HA- and panKChIP-antibodies to detect the respective proteins (arrows). C. Immunoprecipitations (IP) were performed with panKChIP-antibody (WB: HA-antibody) and with HA-antibody (WB: pan KChIP-antibody; note the absence of a KChIP IP signal with homomeric expression of Kv4.2[A14K] constructs in lanes 3 and 6, and faint KChIP IP signals with mixtures of Kv4.2[A14K] and Kv4.2[wt] constructs in lanes 1 and 4, which may have originated from wild-type/A14K heteromers or a small fraction of wild-type homomers).
(PDF)

S2 Fig Subcellular fluorescence detection of epitope-tagged Kv4.2 protein. Kv4.2[wt]^HA,EGFP^ was expressed in CHO cells (see S1 Methods) and detecetd by its EGFP self-fluorescence (EGFP) and surface HA-immunostaining (α-HA surface). A. Fluorescence images showing EGFP (A_1_), α-HA surface (A_2_) and the corresponding overlay (A_3_). B. Bright field microscopy picture showing the transfected fluorescent cell (right) and an untransfected cell (left). Line segment (70 μm) used for intensity profiling is indicated. C. Relative fluorescence intensity profile for Kv4.2[wt]^HA,EGFP^ (normalized to the maximum intensity) showing EGFP (green) and α-HA surface (red).
(PDF)

S3 Fig Functional expression of epitope-tagged Kv4.2 channels. To test if the Kv4.2^HA,EGFP^ channel constructs are functional and display known effects when coexpressed with KChIP2, we expressed them in CHO cells and conducted whole-cell patch-clamp experiments (see S1 Methods). A. Representative currents mediated by Kv4.2[A14K]^HA,EGFP^, Kv4.2[wt]^HA,EGFP^ and Kv4.2[wt]^HA,EGFP^ + KChIP2. Voltage protocol shown below traces. B. Current densities in CHO cells expressing Kv4.2[A14K]^HA,EGFP^, Kv4.2[wt]^HA,EGFP^ or Kv4.2[wt]^HA,EGFP^ + KChIP2. Current densities are similar for the two Kv4.2 constructs in the absence of KChIP2 and potentiated for Kv4.2[wt]^HA,EGFP^ when KChIP2 was coexpressed (§, Student’s t-test).
(PDF)

S4 Fig I_SA_ measurements in the whole-cell and nucleated-patch configuration. With some hippocampal neurons recordings in the nucleated patch-configuration (see S1 Methods) were performed. A. I_SA_ component obtained from a control neuron (expressing only EGFP) with the prepulse-inactivation-subtraction protocol in the whole-cell configuration (left) and after excision of a nucleated patch (right). B. Pairs of I_SA_[whole-cell] and I_SA_[nucleated-patch] for 6 control neurons. Mean values were obtained for I_SA_[whole-cell] (C), I_SA_[nucleated-patch] (D) and the ratio I_SA_[nucleated-patch] / I_SA_[whole-cell] in % (E), for control neurons (grey bars), and for neurons expressing either Kv4.2[wt]^HA,EGFP^ or the dendritc transport mutant Kv4.2[600Δ]^HA,EGFP^ ([32]; black bars). Note that I_SA_[whole-cell] significantly differs from control for wild-type but not for the 600Δ mutant, whereas I_SA_[nucleated-patch] is not significantly different from control for either Kv4.2 construct; * significantly different from control with 0.0001 ≤ p < 0.05; # significantly different from Kv4.2[wt]^HA,EGFP^ with 0.0001 ≤ p < 0.05 (one-way ANOVA); n.s. no significant differences found.
(PDF)

S1 Table Data summary for relative fluorescence intensity and current measurements. (PDF)

S2 Table Data summary for the currents acquired in the AMPA experiments. (PDF)

S1 Methods Experimental procedures used to obtain the data for supporting information. Western blot analysis, immunoprecipitation experiments, immunocytochemistry and electrophysiological recordings were performed with transfected Chinese hamster ovary (CHO) cells, and with some hippocampal neurons electrophysiological recordings were conducted in the nucleated-patch configuration.
(PDF)
