# Supplementary figures and images for: Somatodendritic surface expression of epitope-tagged and KChIP binding-deficient Kv4.2 channels in hippocampal neurons
Source: PLoS One. 2018 Jan 31;13(1):e0191911. doi: 10.1371/journal.pone.0191911 (PMC5792006; doi:10.1371/journal.pone.0191911)

Prechtel et al., S1 Fig

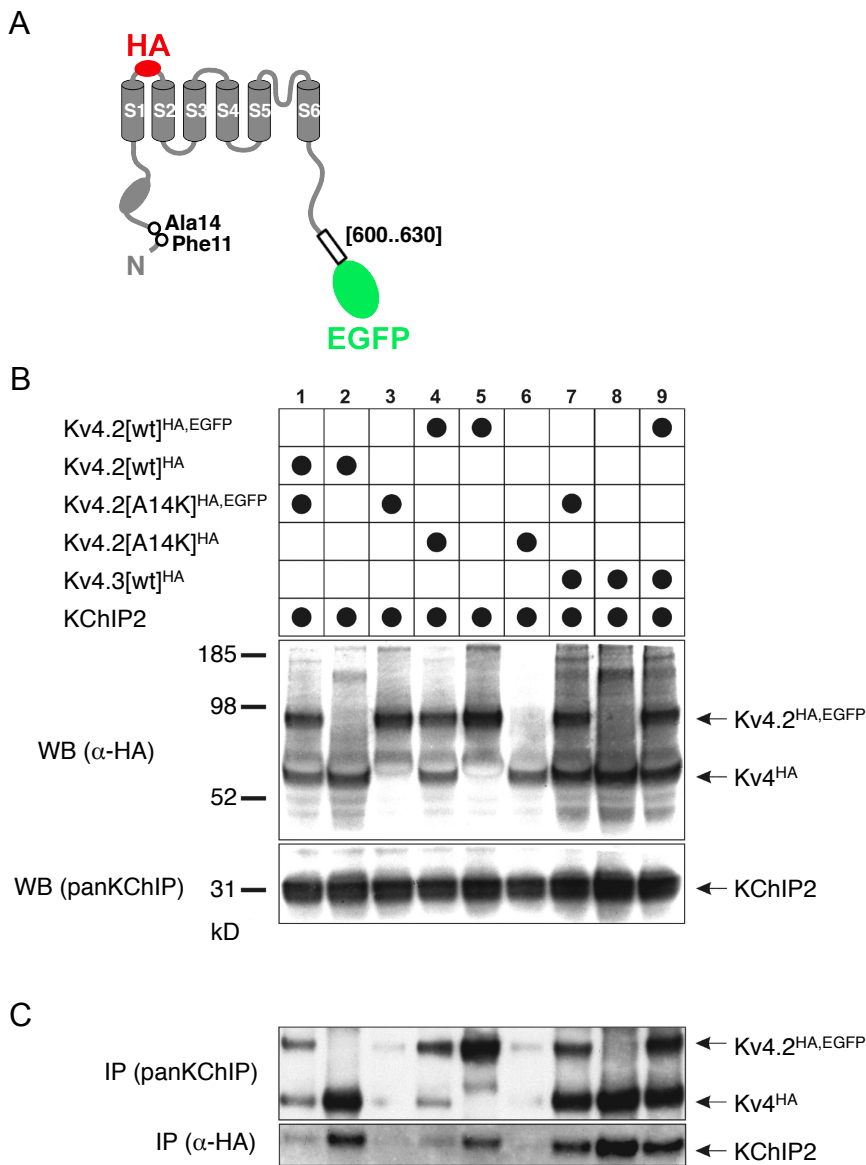

Supplement: S1 Fig — Kv4.2HA,EGFP, Kv4.2HA and Kv4.3HA constructs were coexpressed with KChIP2 in Chinese hamster ovary (CHO) cells in different combinations and detected with HA- and panKChIP-antibodies (see S1 Methods). A. Membrane topology model of the Kv4.2HA,EGFP α-subunit. External HA-tag inserted between transmembrane segments S1 and S2 and EGFP attached to the C-terminal end. Phenylalanine (Phe) 11 and alanine (Ala) 14 near the cytoplasmic N-terminus, and the C-terminal region between amino acid residue 600 and 630, relevant for dendritic transport [32], are indicated. B. Transfected cDNAs (lanes 1–9) and corresponding Western blot analysis (WB) with HA- and panKChIP-antibodies to detect the respective proteins (arrows). C. Immunoprecipitations (IP) were performed with panKChIP-antibody (WB: HA-antibody) and with HA-antibody (WB: pan KChIP-antibody; note the absence of a KChIP IP signal with homomeric expression of Kv4.2[A14K] constructs in lanes 3 and 6, and faint KChIP IP signals with mixtures of Kv4.2[A14K] and Kv4.2[wt] constructs in lanes 1 and 4, which may have originated from wild-type/A14K heteromers or a small fraction of wild-type homomers). (PDF) [file pone.0191911.s001.pdf]

Prechtel et al., S2 Fig

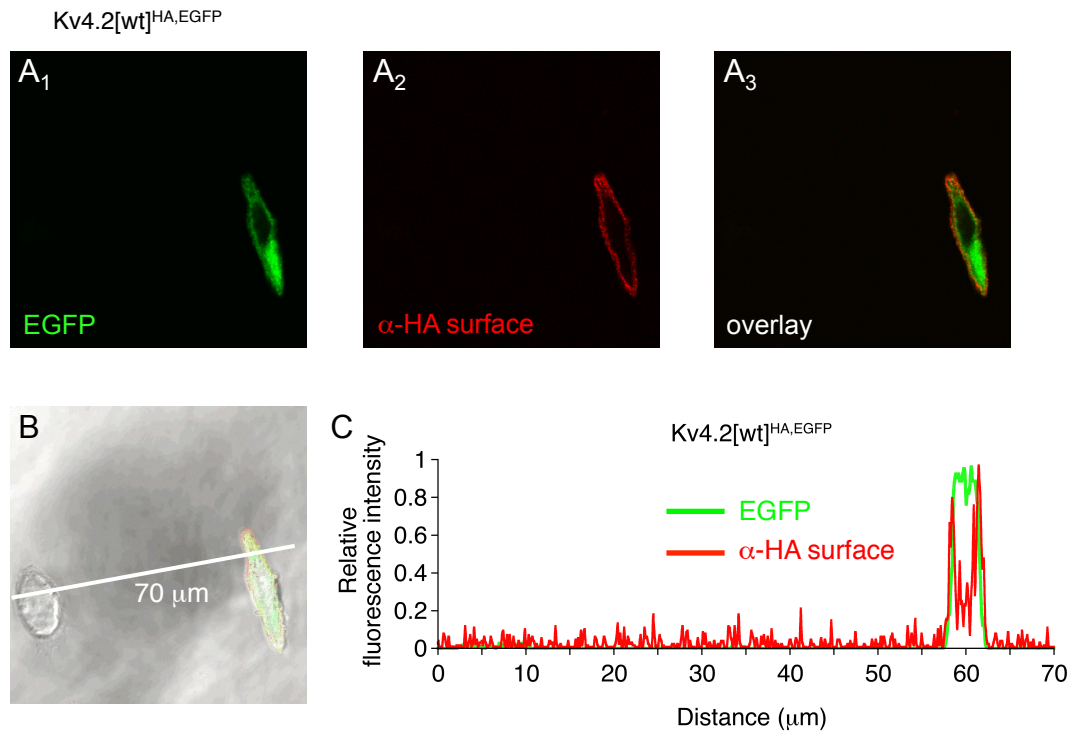

Supplement: S2 Fig — Kv4.2[wt]HA,EGFP was expressed in CHO cells (see S1 Methods) and detecetd by its EGFP self-fluorescence (EGFP) and surface HA-immunostaining (α-HA surface). A. Fluorescence images showing EGFP (A1), α-HA surface (A2) and the corresponding overlay (A3). B. Bright field microscopy picture showing the transfected fluorescent cell (right) and an untransfected cell (left). Line segment (70 μm) used for intensity profiling is indicated. C. Relative fluorescence intensity profile for Kv4.2[wt]HA,EGFP (normalized to the maximum intensity) showing EGFP (green) and α-HA surface (red). (PDF) [file pone.0191911.s002.pdf]

Prechtel et al., S3 Fig

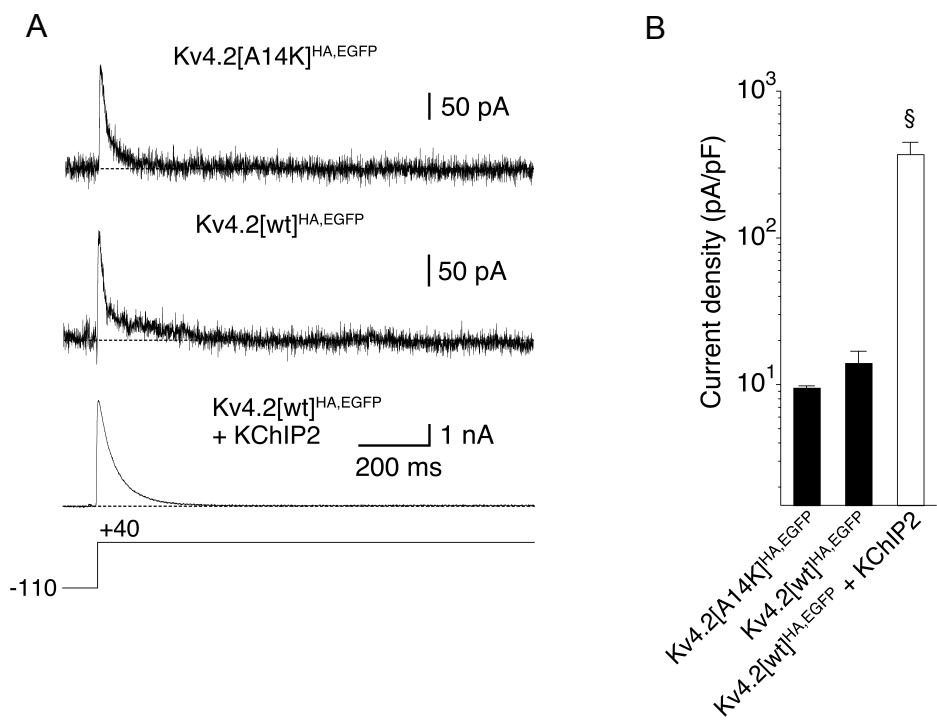

Supplement: S3 Fig — To test if the Kv4.2HA,EGFP channel constructs are functional and display known effects when coexpressed with KChIP2, we expressed them in CHO cells and conducted whole-cell patch-clamp experiments (see S1 Methods). A. Representative currents mediated by Kv4.2[A14K]HA,EGFP, Kv4.2[wt]HA,EGFP and Kv4.2[wt]HA,EGFP + KChIP2. Voltage protocol shown below traces. B. Current densities in CHO cells expressing Kv4.2[A14K]HA,EGFP, Kv4.2[wt]HA,EGFP or Kv4.2[wt]HA,EGFP + KChIP2. Current densities are similar for the two Kv4.2 constructs in the absence of KChIP2 and potentiated for Kv4.2[wt]HA,EGFP when KChIP2 was coexpressed (§, Student’s t-test). (PDF) [file pone.0191911.s003.pdf]

Prechtel et al., S4 Fig

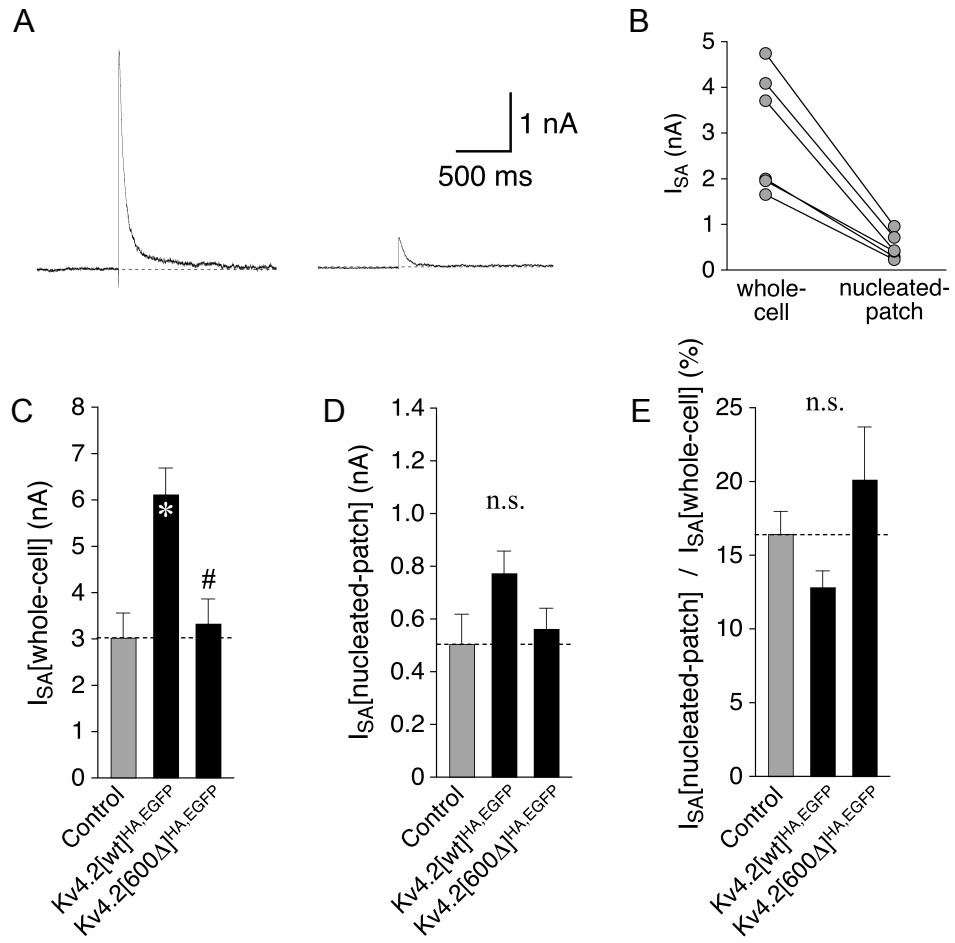

Supplement: S4 Fig — With some hippocampal neurons recordings in the nucleated patch-configuration (see S1 Methods) were performed. A. ISA component obtained from a control neuron (expressing only EGFP) with the prepulse-inactivation-subtraction protocol in the whole-cell configuration (left) and after excision of a nucleated patch (right). B. Pairs of ISA[whole-cell] and ISA[nucleated-patch] for 6 control neurons. Mean values were obtained for ISA[whole-cell] (C), ISA[nucleated-patch] (D) and the ratio ISA[nucleated-patch] / ISA[whole-cell] in % (E), for control neurons (grey bars), and for neurons expressing either Kv4.2[wt]HA,EGFP or the dendritc transport mutant Kv4.2[600Δ]HA,EGFP ([32]; black bars). Note that ISA[whole-cell] significantly differs from control for wild-type but not for the 600Δ mutant, whereas ISA[nucleated-patch] is not significantly different from control for either Kv4.2 construct; * significantly different from control with 0.0001 ≤ p < 0.05; # significantly different from Kv4.2[wt]HA,EGFP with 0.0001 ≤ p < 0.05 (one-way ANOVA); n.s. no significant differences found. (PDF) [file pone.0191911.s004.pdf]
