# Supplementary material for: Somatodendritic surface expression of epitope-tagged and KChIP binding-deficient Kv4.2 channels in hippocampal neurons
Source: PLoS One. 2018 Jan 31;13(1):e0191911. doi: 10.1371/journal.pone.0191911 (PMC5792006; doi:10.1371/journal.pone.0191911)
Supplement: S1 Table — (PDF) [file pone.0191911.s005.pdf]

Prechtel et al., S1 Table

Data summary for relative fluorescence intensity and current measurements

	α-HA surface / EGFP (%)	n	I_{SA} (nA)	I_D (nA)	I_{SA} / I_D	n
Control	-		3.01 ± 0.49	3.08 ± 0.28	0.891 ± 0.083	22
KChIP2	-		3.84 ± 0.26	3.96 ± 0.18	0.974 ± 0.052	20
$Kv4.2[wt]^{HA,EGFP}$	52 ± 4	21	5.81 ± 0.52 *	3.39 ± 0.30	1.751 ± 0.096 **	21
$Kv4.2[A14K]^{HA,EGFP}$	53 ± 5	21	5.48 ± 0.63 *	3.79 ± 0.35	1.416 ± 0.081 *	15
$Kv4.2[wt]^{HA,EGFP}$ + KChIP2	69 ± 5 §	20	7.07 ± 0.49 **	3.87 ± 0.26	1.902 ± 0.131 **	28
$Kv4.2[A14K]^{HA,EGFP}$ + KChIP2	50 ± 4	20	5.96 ± 0.51 *	4.35 ± 0.39 *	1.505 ± 0.118 *	20

Fluorescence intensity integral ratios (α-HA surface / EGFP), I_{SA} and I_D amplitudes and I_{SA} / I_D ratios; § significantly different from the value obtained in the absence of coexpressed exogenous KChIP2; * significantly different from control with $0.0001 \leq p < 0.05$; ** significantly different from control with $p < 0.0001$; one-way ANOVA.
