# Supplementary material for: Somatodendritic surface expression of epitope-tagged and KChIP binding-deficient Kv4.2 channels in hippocampal neurons
Source: PLoS One. 2018 Jan 31;13(1):e0191911. doi: 10.1371/journal.pone.0191911 (PMC5792006; doi:10.1371/journal.pone.0191911)
Supplement: S2 Table — (PDF) [file pone.0191911.s006.pdf]

Prechtel et al., S2 Table

Data summary for the currents acquired in the AMPA experiments.

	I_{SA} (nA)	I_D (nA)	I_{SA} / I_D	n
Control				
**untreated**	3.90 ± 0.52	2.79 ± 0.53	1.945 ± 0.363	12
**AMPA**	2.56 ± 0.44	1.95 ± 0.39	1.587 ± 0.176	15
Kv4.2[wt]^{HA,EGFP} + KChIP2				
**untreated**	6.40 ± 0.75	0.96 ± 0.08	7.718 ± 1.235	14
**AMPA**	2.31 ± 0.21 **	0.96 ± 0.31	3.512 ± 0.411 *	16
Kv4.2[A14K]^{HA,EGFP} + KChIP2				
**untreated**	4.10 ± 0.46	0.76 ± 0.08	5.880 ± 0.686	14
**AMPA**	2.66 ± 0.20 *	0.81 ± 0.09	4.505 ± 1.021	16

I_{SA} and I_D amplitudes and I_{SA} / I_D ratios; * significantly different from untreated with $0.0001 \leq p < 0.05$; ** significantly different from untreated with $p < 0.0001$; unpaired Student's t-test.
