# Supplementary material for: Somatodendritic surface expression of epitope-tagged and KChIP binding-deficient Kv4.2 channels in hippocampal neurons
Source: PLoS One. 2018 Jan 31;13(1):e0191911. doi: 10.1371/journal.pone.0191911 (PMC5792006; doi:10.1371/journal.pone.0191911)
Supplement: S1 Methods — Western blot analysis, immunoprecipitation experiments, immunocytochemistry and electrophysiological recordings were performed with transfected Chinese hamster ovary (CHO) cells, and with some hippocampal neurons electrophysiological recordings were conducted in the nucleated-patch configuration. (PDF) [file pone.0191911.s007.pdf]

## Prechtel et al., S1 Methods

Where not otherwise stated, S1 Methods comply with Materials and methods.

### Chinese hamster ovary (CHO) cell culture and transfection

CHO cells were cultured as described previously (Bähring et al., 2001; Callsen et al., 2005). The cells were plated at a density of 5 x $10^4$ per 50 mm dish for Western blot and immunoprecipitation experiments, and 3 - 4 x $10^4$ per 35 mm dish for electrophysiological experiments. Transfections were performed one day after plating using Lipofectamine 2000 (Roche) according to the manufacturer's protocol, as described previously (Bähring et al., 2001; Callsen et al., 2005). The cells were used for the experiments on the next two days (see below).

### Western blot and immunoprecipitation experiments

CHO cells were transfected with Kv4.$2^{HA,EGFP}$, Kv4.$2^{HA}$, Kv4.$3^{HA}$ and KChIP2 cDNAs (each 2 μg per culture dish) in different combinations (see S1 Fig). The transfected cells were washed with PBS and harvested in lysis buffer containing 150 mM NaCl, 50 mM HEPES, 2 mM EDTA, 0.5% Triton X-100, pH 7.4 NaOH, 10 μl protease inhibitor cocktail (Sigma) per ml, and incubated for 1.5 h at 4 °C. After pelleting the cellular debris (14000 rpm, 15 min, 4 °C) equal amounts of supernatant were separated by SDS-PAGE and transferred to nitrocellulose membranes. After a blocking reaction (PBS with 0.05% Tween and 5% non-fat milk powder) the membranes were incubated with primary antibody (rat anti-HA or rabbit panKChIP 1:500, overnight, 4 °C). After washing the membranes with PBS, they were incubated with horseradish peroxidase (HRP)-conjugated secondary antibody (anti rat HRP from goat, Dianova, 1:1000; anti rabbit HRP from goat, Vector Laboratories, 1:5000) for 1 h at RT. Enhanced chemiluminescence reagents were used for signal detection, and the data were quantified with Quantify One (Bio-Rad). For immunoprecipitation experiments primary antibodies were incubated with proteinG Dynabeads (Dynal, Invitrogen). Equal amounts of protein were immunoprecipitated with Dynabeads-coupled panKChIP- or HA-antibody (overnight, 4 °C). The immunoprecipitates were washed with lysis buffer and eluated with Nupage sample buffer (Invitrogen) under reducing conditions (12 min, 70 °C). With the supernatant, Western blot analysis was performed as described (see S1 Fig).

**Immunocytochemistry**

CHO cells were transfected with 2 μg Kv4.2[wt]$^{HA,EGFP}$ cDNA per well (see S2 Fig).

**Electrophysiological experiments**

CHO cells were transfected with 0.1 μg Kv4.2$^{HA,EGFP}$ cDNA in the absence or presence of 1 μg KChIP2 cDNA per culture dish. For the recordings the cells were superfused with a solution containing (in mM) 135 NaCl, 5 KCl, 2 $CaCl_2$, 2 $MgCl_2$, 5 HEPES, 10 sucrose (pH 7.4, NaOH), and patch-pipettes were filled with a solution containing (in mM) 125 KCl, 1 $CaCl_2$, 1 $MgCl_2$, 11 EGTA, 10 HEPES, 10 sucrose, 2 glutathione, 2 $K_2$-ATP (pH 7.2, KOH). The holding voltage was -80 mV, outward currents were activated by voltage pulses to +40 mV from a prepulse voltage of -110 mV, and a P/3 protocol was used for leak subtraction. Current densities were calculated based on the whole-cell capacitance (see S3 Fig). With some neurons recordings were made in the nucleated-patch configuration, where a large vesicle containg the nucleus is excised (Sather et al., 1992). To favor nucleated-patch formation and stability (Sather et al., 1992) the solutions used in these experiments differed from the ones used for our conventional neuronal whole-cell recordings: The neurons were superfused with a solution containing (in mM) 140 NaCl, 2.8 KCl, 1 $CaCl_2$, 10 HEPES (pH 7.2 NaOH), and the hypoosmotic and divalent-free pipette solution contained (in mM) 98 KCl, 7 HEPES and 7 EGTA (pH 7.2, KOH).

**Refernces**


1. Bähring R, Dannenberg J, Peters HC, Leicher T, Pongs O, Isbrandt D. Conserved Kv4 N-terminal domain critical for effects of Kv channel-interacting protein 2.2 on channel expression and gating. J Biol Chem. 2001;276: 23888-23894.

2. Callsen B, Isbrandt D, Sauter K, Hartmann LS, Pongs O, Bähring R. Contribution of N- and C-terminal Kv4.2 channel domains to KChIP interaction. J Physiol. 2005;568:397-412. PubMed PMID: 16096338

3. Sather W, Dieudonné S, Macdonnald JF, Ascher P. Activation and desensitization of N-methyl-D-aspartate receptors in nucleated outside-out patches from mouse neurons. J Physiol. 1992; 450: 643-672.
